# Effect of landscape features on the relationship between *Ixodes ricinus* ticks and their small mammal hosts

**DOI:** 10.1186/s13071-016-1296-9

**Published:** 2016-01-15

**Authors:** Grégoire Perez, Suzanne Bastian, Albert Agoulon, Agnès Bouju, Axelle Durand, Frédéric Faille, Isabelle Lebert, Yann Rantier, Olivier Plantard, Alain Butet

**Affiliations:** UMR 6553 Ecosystème, Biodiversité, Evolution, Centre National de la Recherche Scientifique—Université de Rennes 1, Avenue du Général Leclerc, Rennes, 35042 France; UMR1300 Biologie, Epidémiologie et Analyse de Risque, Institut National de la Recherche Agronomique—LUNAM Université, Oniris, Ecole Nationale Vétérinaire, Agroalimentaire et de l’Alimentation Nantes-Atlantique, Atlanpole, la Chantrerie, Nantes, 44307 France; UR0346 Epidémiologie Animale, Institut National de la Recherche Agronomique, Route de Theix, Saint-Genès-Champanelle, 63122 France

**Keywords:** Agricultural landscape, Forest landscapes, *Ixodes ricinus*, *Apodemus sylvaticus*, *Myodes glareolus*, Ecotone, Host-parasite system

## Abstract

**Background:**

The consequences of land use changes are among the most cited causes of emerging infectious diseases because they can modify the ecology and transmission of pathogens. This is particularly true for vector-borne diseases which depend on abiotic (*e.g*. climate) and biotic conditions (*i.e*. hosts and vectors). In this study, we investigated how landscape features affect the abundances of small mammals and *Ixodes ricinus* ticks, and how they influence their relationship.

**Methods:**

From 2012 to 2014, small mammals and questing *I. ricinus* ticks were sampled in spring and autumn in 24 sites located in agricultural and forest landscapes in Brittany, France. We tested the effects of landscape features (composition and configuration) on the abundances of small mammal species and immature ticks and their relationship. Additionally, we quantified the larval tick burden of small mammals in 2012 to better describe this relationship.

**Results:**

The nymph abundance was positively influenced by the larval occurrence and the wood mouse *Apodemus sylvaticus* abundance the previous spring because they hosted tenfold more larvae than the bank vole *Myodes glareolus*. The bank vole abundance in spring and autumn had a negative and positive effect, respectively, on the nymph abundance. In agricultural landscapes, wood mice were positively influenced by woodland cover and woodland/hedgerow-grassland ecotone, whereas bank voles showed the opposite or non-significant responses to these landscape variables. The woodland cover had a positive effect on immature ticks.

**Conclusion:**

The landscape configuration, likely by affecting the landscape connectivity, influences the small mammal communities in permanent habitats. Our study showed that the wood mouse, due to its dominance and to its tolerance to ticks, feeds a substantial proportion of larvae. The acquired resistance to ticks in the bank vole can reduce its role as a trophic resource over time. The nymph abundance seems indirectly influenced by landscape features via their effects on the small mammal community. To enhance our understanding of the epidemiology of tick-borne diseases within landscapes, further studies will integrate data on pathogen prevalence and investigate explicitly the effect of landscape connectivity on host-vector-pathogen systems.

## Background

The consequences of land use changes and agricultural intensification are among the most cited causes of emerging diseases in the last decades [1, 2]. Indeed, the recent evolution of agricultural landscapes is a major driver of the modification of vertebrate and invertebrate communities [3, 4], but its influence on the risk of vector-borne diseases has been scarcely studied at the landscape scale (but see [5] for a review of landscape epidemiology of vector-borne diseases).

The complex relationship between ticks and their hosts is a good system for investigating interactions between parasite and host populations in heterogeneous landscapes. The ticks (Ixodida) are obligate hematophagous arachnids and vectors of many infectious diseases (e.g. Lyme disease, piroplasmosis, and tick-borne encephalitis [6]) that can affect vertebrate host populations, including humans, livestock, and wildlife. The hard ticks (Ixodidae) are particularly interesting because each immature stage (larvae and nymphs) needs to take a single blood meal to molt into the next stage and female ticks need to take a single blood meal to produce their eggs. Thus, during their lifecycle, they take only two (for males) or three (for females) blood meals, spaced by several months depending on temperature and relative humidity [7, 8]. As ticks spend most of their lifecycle off their hosts, their survival and development depend on the environmental conditions to which they are exposed.

The parasite-host-habitat relationship is especially complex for exophilic ticks (with active host seeking) like *I. ricinus*, which is the most common tick species in Western Europe. *I. ricinus* has a wide range of hosts. Larvae can feed on many different vertebrate host species including mammals, birds and lizards. Small mammals are believed to be the most important hosts because they are numerous and move close to the ground (where larval ticks quest) [9–11]. Nymphal ticks feed preferentially on birds and medium to large mammals and adult female ticks feed preferentially on larger mammals such as ungulates, for instance roe deer *Capreolus capreolus* and cattle [12–15]. Nevertheless, small mammals can also feed nymphs and exceptionally adult female ticks [16], from which they can acquire tick-borne infectious agents, as they have been shown to be reservoir hosts, for instance, for *Borrelia burgdorferi* sl., *Anaplama phagocytophilum* and *Babesia microti* (respectively, two bacteria and a protozoan responsible for Lyme disease, anaplasmosis and piroplasmosis). As most small mammal populations display inter-annual fluctuations, this potentially leads to outbreaks of nymphal tick populations the year after high small mammal abundance. Such time-lagged relationships between small mammal abundances and exophilic nymphal tick abundances have been shown previously, but these studies focused mainly on homogeneous forest landscapes [17–19]. Indeed, changes in small mammal communities can influence tick-borne disease transmission in different ways.

First, small mammal species differ in their competence for transmitting tick-borne pathogens [20–22]. For instance, the bank vole *Myodes glareolus* seems to be a more competent reservoir for *B. microti*, a blood parasitic protozoan that may cause human piroplasmosis, than the wood mouse *Apodemus sylvaticus* [22]. In contrast, the wood mouse seems to yield more ticks infected with *Borrelia afzelii*, a rodent-specific genospecies of the complex of bacteria responsible for Lyme disease [23, 24]. The host community composition can affect tick-borne disease transmission via the “dilution effect”, which predicts that decreased biodiversity can increase disease transmission [25, 26]. A high abundance of one reservoir species can amplify the infectious agents for which it is most competent, while the abundance of incompetent hosts for these infectious agents can reduce their transmission.

Population dynamics and community structure of small mammal hosts are influenced by landscape features [27, 28], which can therefore have consequences on the tick populations and the transmission of tick-borne pathogens [12, 23, 29]. In North America, woodland fragmentation (as estimated by the reduction in wood size) increased the prevalence of *B. burgdorferi* sensulato (s.l.) [30]. The authors’ explanation is that white-footed mice *Peromyscus leucopus*, which are important reservoirs of the bacteria, are favoured by woodland fragmentation [30–32]. Small mammal species differ in their resistance to ticks. For instance, repeated infestation of bank voles by larval ticks reduced the feeding success of larval ticks, whereas this was not the case for wood mice [22, 23, 33]. This difference in tick resistance can affect the relative contribution of each small mammal species as a source of blood meals for ticks [23]. Small mammal species also differ in their exposure to ticks because of differences in home range and behaviour [34, 35].

Landscape features have evolved drastically in recent decades in agricultural landscapes (i.e. field enlargement, hedgerow removals). Changes in agricultural landscapes also affect the communities of small mammals [27]. Small mammals are considered as primary hosts of *I. ricinus* larvae and therefore as a determinant of the abundance of questing nymphs, the most important stage from an epidemiological perspective (abundant and possibly infected during their first blood meal). However, to our knowledge, few studies have considered the effects of the composition (amount of land cover type) and configuration (structure of land cover type) of agricultural landscapes on the interactions between habitats, ticks and their hosts (nevertheless, for interesting approaches see [36, 37]). A better understanding of the role of small mammal communities as trophic resources for ticks and potential reservoirs of pathogens is thus necessary for the prevention of many tick-borne diseases.

The aim of this study was to evaluate the relationship between the abundance of small mammals and the abundance of questing *I. ricinus* nymphs, and the role of landscape features on this relationship. Abundances of small mammals and ticks were compared along a gradient of landscape and land use features. Deciduous forest landscapes provided reference conditions as wooded habitats are considered favorable for ticks because temperature and humidity are buffered. Data on the abundances of small mammals and immature *I. ricinus* ticks were collected over a period of 3 years, in late spring and early autumn, when peak populations of small mammals and peak activity of ticks interact most strongly [7, 38–41].

As larvae mainly disperse by attaching to hosts and as small mammal hosts have small home ranges, we assumed that the abundance of questing nymphs in a given year was related to the abundance of larvae that fed on the hosts in the same area the previous year. We hypothesized that: (1) The occurrence of *I. ricinus* larvae drives the abundance of *I. ricinus* nymphs the following year. (2) The abundance of small mammals drives the abundance of *I. ricinus* nymphs the following year. (3) The contribution of each small mammal species to the abundance of *I. ricinus* nymphs depends on their intrinsic susceptibility to tick infestation, their habitat preference, and the landscape features. (4) Landscape features with buffered humidity and temperature, like woodland areas and wooded habitats-grassland ecotones, will be positively related to tick density because they act as habitats for ticks and their hosts.

We first investigated the influence of landscape features on small mammal abundances, the larval occurrence and the questing nymph abundance. Then, we tested the effects of small mammal abundances and the larval occurrence on the questing nymph abundance the following year. We examined the underlying mechanisms by testing the relationship between larval occurrence and the larval burden on small mammals and the relationship between the larval burden on small mammals and the questing nymph abundance the following year. Finally, we built integrative models to estimate the relative effects of larval occurrence, small mammal abundances, and landscape features on the questing nymph abundance.

## Methods

### Study area and sampling periods

The study area belongs to the “Zone atelier Armorique”, a labeled LTER (Long Term Ecological Research) area of the CNRS (Centre National de la Recherche Scientifique) where ecological studies have been conducted for over 20 years. This area is located in the north-east of the Brittany region (France), south of the Mont-Saint-Michel Bay (48°30’ N, 1°32’ W; Appendix 1). This study area has three different landscapes that were sampled in this study: (1) a crop-dominated landscape with few hedgerows (North of the area), (2) a traditional mixed crop-livestock landscape with a dense hedgerow network (South-West of the area) and (3) a managed deciduous forest of about 1000 ha (South-East of the area). Because tick activity is mainly bimodal (April-June and September-October) under temperate-oceanic climate conditions [38, 39, 41] and because most small mammals start breeding in spring and their abundance peaks in autumn [42], we trapped small mammals and sampled ticks in May-June and October, during years 2012, 2013 and 2014 (except autumn 2014 for ticks for logistical reasons).

In the study there were 24 sampling sites that were distributed across the three landscape types as follows (see figure in Appendix 1): in the crop-dominated landscape there were 3 sites along hedgerows and 3 sites along wood edges; in the traditional mixed crop-livestock landscape there were 3 sites along hedgerows and 3 sites along wood edges; in the forest landscape there were 6 sites along the forest edge and 6 sites located in the forest core. 18 out of the 24 trapping sites (all except the 6 sites located in the forest core) were bordered by a meadow. These 18 sites therefore contained a transition (ecotone) between meadow and wooded habitat (hedgerow, wood edges, or forest edges). This ecotone between meadow and wooded habitat is considered an optimal habitat for both ticks and small mammals [43]. To avoid spatial autocorrelation, all 24 sites were separated from each other by a distance of at least 500 metres. These 24 sampling sites were used for both small mammal and tick.

### Sampling of small mammals and attached ticks

The abundances of small mammals were estimated for each trapping session by using 100 metre-long trap-lines of 34 INRA live-traps with dormitory boxes. Traps were spaced every 3 meters and baited with pieces of apple and a mixture of dry cat food and seeds for domestic rodents [42, 44, 45]. The traps were checked in the morning, after they had been in place for 24 h, and they were retrieved after 48 h. This resulted in 68 trap-nights per trap-line and an overall trapping effort of 1632 trap-nights each season. Trapped small mammals were brought back to our field laboratory, identified to the species level, and euthanized. In 2014, animals were marked and kept alive for another study and were released after the end of the trapping session and at least 500 m away from all the capture sites to avoid recapture. The data from 2014 were used to analyse the effect of the landscape on the abundance of small mammals, but these data were not used in the analysis of the small mammal-tick relationships. As the sampling effort was the same for each site and season, we used the total number of captured animals per trap-line as an index of the true small mammal abundance [46].

In 2012, ectoparasites were removed from small mammals and conserved in 70 % ethanol, either immediately or after deep-frozen storage (−20 °C) of the carcasses. Ticks were identified to stage (larva, nymph or adult) and species using a binocular microscope and the identification key of Pérez-Eid [47]. We identified two-thirds of the larvae and they all belonged to the same species, *I. ricinus*. We therefore assumed that all larvae belonged to this species and that all other tick species were rare.

### Ethics statement

The animals trapped in this experiment were not protected species. The traps that were used did not stress or harm the animals. All individuals in 2012 and 2013 were euthanized by authorized experimenters according to current French law and the European guidelines on the use of animals in science. In 2014, all the animals were released at the end of the trapping session.

### Sampling of questing ticks

Ticks were sampled at the 24 sites along 300-metre transects that contained ten sub-transects. A sub-transect consisted of applying the “dragging method” along 10 metres, for a sampling area of 10 m^2^. The “dragging method” consists of pulling a 1-m^2^ white flannel blanket along the ground at a speed of about 0.5 metres per second [48, 49]. Sub-transects were spaced 20 metres apart (to represent all microclimate variations in one site and to ensure the independence of sub-transects for another study at a finer scale). Thus each transect covered a total area of 100 m^2^. For the 12 plots that were at ecotones, either at the wood-meadow interface (*n* = 6 plots) or at the forest-meadow interface (*n* = 6 plots), we set two paired transects: one in the wood or the forest and one in the adjacent meadow. Thus there were a total of 36 transects for sampling tick abundance: 6 transects along hedgerows, 2 × 6 transects at the edge of woods, 6 transects in the forest core and 2 × 6 transects at the forest edge. All tick samplings were conducted when the ground was reasonably dry, i.e*.* we avoided sampling after rain or when there was morning dew.

After each sub-transect, all ticks attached to the cloth were removed and placed in individual micro tubes containing 70 % ethanol. Species and stage identification of ticks was done as described previously for the attached ticks [47]. We used the total number of questing *I. ricinus* nymphs per transect (100 m^2^) as an index of the abundance of questing *I. ricinus* nymphs. For larvae, the number of sub-transects with at least one larva were used as an index of recruitment (as it shows that at least one female tick laid her eggs at this location). This index ranged from 0 to 10 (0: no sub-transect with at least one larva and 10: there was at least one larva for each of the ten sub-transects; for a similar approach see [44]) and will hereafter be referred to as the “larval occurrence index”. For paired transects, we chose to use the mean of the two transects in the subsequent analyses because it better described the local questing nymph abundance.

### Landscape variables

To analyse the spatial heterogeneity of the landscape surrounding the sampling plots, we used a polygon shape file of land cover provided by the “Zone atelier Armorique”. This shape file was created by interpretation of aerial ortho-photographs taken in summer 2012 with a resolution of 0.5 metres. The variables were computed directly in ArcGIS 10.1 (ESRI®) or in Fragstat 4.2.598 [50] after conversion from vector (polygon shape file) to raster format with a cell size of 0.5 metres. We calculated the variables in 250-metre “radius” buffers centered on trapping lines. We chose a distance of 250 metres to avoid the superposition of the buffers and because this distance is larger than the home range of the wood mouse, which was the most mobile species trapped in our study [46, 51, 52]. The landscape contained forest tracks, which consist of bare soil covered by dead leaves or herbaceous plants and frequented only by pedestrians, bikers, equestrians, and working machines. We considered these forest tracks as wooded habitat. We verified that wooded patches were not artificially divided into two or more patches by the buffers to avoid confounding the measure of distance from one wooded patch to another. We corrected wooded patch structure by joining artificially the two parts of the original wooded patches by a wooded strip (one case). When only one wooded patch was present in the buffer, we used the distance of the “radius” of the buffers (250 metres).

We extracted the following six variables : (1) the length of ecotones between wooded habitat and grassland (“EcoL”), (2) the proportion of woodland cover (“Wood”), (3) the proportion of grassland cover (“Grass”), (4) the proportion of crops (“Crops”), (5) the mean distance between wooded patches (“ENN-Wood”), (6) the perimeter-area ratio of wooded patches (“PARA-Wood”). The variables ENN-Woodand PARA-Wood were weighed by the areas of the wooded patches. Computation details for ENN (Euclidian Nearest-Neighbor distance) and PARA (Perimeter Area RAtio) are given in the Fragstats manual [50].

To avoid collinearity in our analyses, we tested the relationships between the variables with Pearson’s correlation tests. When a significant correlation was found with R^2^ > 0.50, we kept only the more relevant variable. The forest sites with homogeneous woodland cover biased the relationship of this variable with the other explanatory variables. So, when considering only the agricultural landscapes, Wood was negatively related to PARA-Wood (R^2^ = 0.760, *t* = −5.63, *p* < 0.001), while EcoL was positively related to Grass (R^2^ = 0.725, *t* = 5.14, *p* < 10^−3^) and negatively related to Crops (R^2^ = 0.489, *t* = −3.10, *p* = 0.011).

We finally used the following three extracted variables (Table 1): EcoL, Wood, and ENN-Wood. The variables EcoL and Wood are representative of the amount of suitable permanent habitat for small mammals and ticks. The variable EcoL is also an index of landscape connectivity in agricultural landscapes. The variable ENN-Wood is an index of the woodland fragmentation, weakly related to Wood. We chose not to keep the variable Crops because it was too closely related to woodland cover when considering the landscapes together. This variable was also more difficult to interpret because it included various crops.

**Table 1:** Landscape variables considered in the study

Landscape features	Description (unit)	Expected effect on rodent abundance	Expected effect on ticks^a^
EcoL	Wooded habitats/hedgerow-grassland ecotone length (m)	Positive (shelter, food availability, enhanced dispersion through woodland connectivity)	Positive (humidity and temperature, frequented by roe deer, cattle and other hosts)
Wood	Proportion of wooded areas in the buffer (%)	Positive (shelter, food availability)	Positive (humidity and temperature, high density of roe deer and other hosts)
ENN-Wood	Area-weighted^b^ mean distance between nearest edges of wooded patches (m)	Species-dependent: positive (reduced predation/competition) or negative (reduced connectivity: impeded dispersion)	Positive (concentration of roe deer and other hosts in permanent habitats) or negative (lower overall roe deer density)

The landscape variables were extracted in a buffer of 250 metres around the trap-lines. For each landscape variable, we give a definition, the units, and we indicate the expected effects on the abundances of small mammals and *I. ricinus* ticks

^a^ Effect considered independently of small mammal abundances

^b^ In the calculation of the ENN-Wood the values computed for large wooded patches have a greater weight, proportional to their area

### Statistical analyses

#### Checking for spatial autocorrelation and species interactions

To assure the independence of the samples, we controlled for spatial autocorrelation. We computed Moran’s I for small mammal species abundance, questing nymph abundance and larval occurrence in each season [53]. The exclusion of one important host species by another could lead to an indirect effect of the latter on the nymph abundance. To detect potential competition between wood mice and bank voles (the two most frequently trapped species), we tested the relationship between their abundances with linear models on all the data, the data separated by year (2012, 2013 and 2014) or by season (spring and autumn).

#### *Temporal variation of small mammal abundance and* I. ricinus *nymph abundance*

To analyze the temporal variation of small mammal abundances, we compared the mean small mammal abundances between years with Wilcoxon signed rank tests. We compared the average small mammal abundance between spring and autumn with one-tailed paired Wilcoxon signed rank tests (spring < autumn). We compared the questing nymph abundance between years and between seasons with Wilcoxon signed rank tests. To determine whether small mammal species abundances in spring and autumn could be considered independent and whether nymph abundances at spring and autumn could be considered independent, we tested the correlation between small mammal species abundances in spring and autumn and between nymph abundances in spring and autumn by fitting linear models.

#### *Small mammal* I. ricinus *larval burden analyses*

The larval burden on small mammals in 2012 was compared between species for each season and between seasons for the same species with Mann–Whitney tests. These analyses were done only in 2012 because the sample size was too small in 2013 for a reliable analysis (for wood mice and bank voles, respectively: 16 and 12 in spring and 72 and 6 in autumn).

#### *The effect of landscape features on small mammal abundances and questing immature* I. ricinus *ticks*

To test the effect of the landscape features and the sampling year, we modelled each of three response variables: (1) small mammal abundance, (2) *I. ricinus* larval occurrence index, and (3) questing nymph abundance as a function of the extracted landscape variables and the sampling year. The small mammal abundances are count data and the larval occurrence index follows the definition of a Poisson error distribution, we therefore used generalized linear models with a Poisson error distribution (GLMPs) and multiple explanatory variables. We used a negative binomial error distribution (GLMNBs) to account for the over-dispersion of the nymph abundance data [54]. To facilitate the comparison of slopes between variables measured in different units, all landscape variables were scaled to z-scores (mean of zero and a standard deviation of 1). For model simplification, the best model was selected according to AICc (Akaike Information Criterion corrected). To determine the significant variables in the simplified models, we chose an alpha level of 0.05. The *p*-values of the simplified models were calculated by applying a type III ANOVA to the model.

We conducted separate analyses of nymph abundance for the spring and autumn, despite the fact that few nymphs were collected in autumn. Preliminary analysis suggested that the homogeneous woodland cover in the forest sites biased the effects of the landscape features. To better estimate the effects of the landscape features, we therefore re-ran the analysis after excluding the data from the forest sites.

#### S*mall mammal-tick relationships*

To test the small mammal abundances-tick relationships, we modelled the questing nymph abundance in spring as a simple function of each of the following four explanatory variables: (1) small mammal abundance the previous spring, (2) small mammal abundance the previous autumn, (3) larval occurrence index the previous spring, and (4) larval occurrence index the previous autumn. Questing nymph abundance was analysed with GLMNBs to account for the over-dispersion of these data [54].

To determine to what extent tick burden depends on larval occurrence, we used linear models to test the relationship between the larval occurrence index and the host larval burden. To confirm that where small mammals had more larvae, more nymphs were found the following year, we used linear models to test the relationship between host larval burden and the questing nymph abundance the next spring. We used two different measures of host larval burden: the total number of larvae attached to rodents per trap-line and the mean number of larvae per individual rodent per trap-line.

#### *Integrative models to predict the questing* I. ricinus *nymph abundance*

Finally, to discern the more important explanatory variables of nymph abundances, we modelled the questing nymph abundance as a function of (1) landscape variables, (2) the small mammal abundances the previous year, (3) the larval occurrence index the previous year, and (4) the sampling year. We transformed the rodent abundances and the larval occurrence index to z-scores to facilitate comparison of the partial regression coefficients. We transformed these explanatory variables to facilitate comparison of the partial regression coefficients. We used GLMNBs and we selected the best model according to AICc as described above. We conducted separate analyses for the agricultural landscape and the forest landscape to allow for the possibility that the relationships between the explanatory variables (larval occurrence index, small mammal abundances, landscape features) and the response variable (questing nymph abundance) might depend on the landscape.

#### Statistical computing

All analyses were done with R software version 3.1.0 (The R Development Core Team [55]). We used the R packages “ape” for the autocorrelation analyses, “MASS” for the generalized linear models (GLMPs and GLMNBs),”MuMIn” for the GLMMs selection procedure and “car” for the type III ANOVAs.

## Results

### Small mammal abundances

A total of 1145 small mammals were collected, including 876 wood mice *Apodemus sylvaticus* (76.5 %), 255 bank voles *Myodes glareolus* (22.3 %), 6 field voles *Microtus agrestis* (0.52 %), 4 Millet’s shrews *Sorex coronatus* (0.35 %), 3 common pine voles *Microtus subterraneus* (0.26 %), and 1 greater white-toothed shrew *Crocidura russula* (0.09 %). Based on the low abundances of the last four species, only the abundances of the wood mouse and the bank vole were considered for further analysis.

**Fig. 1:**
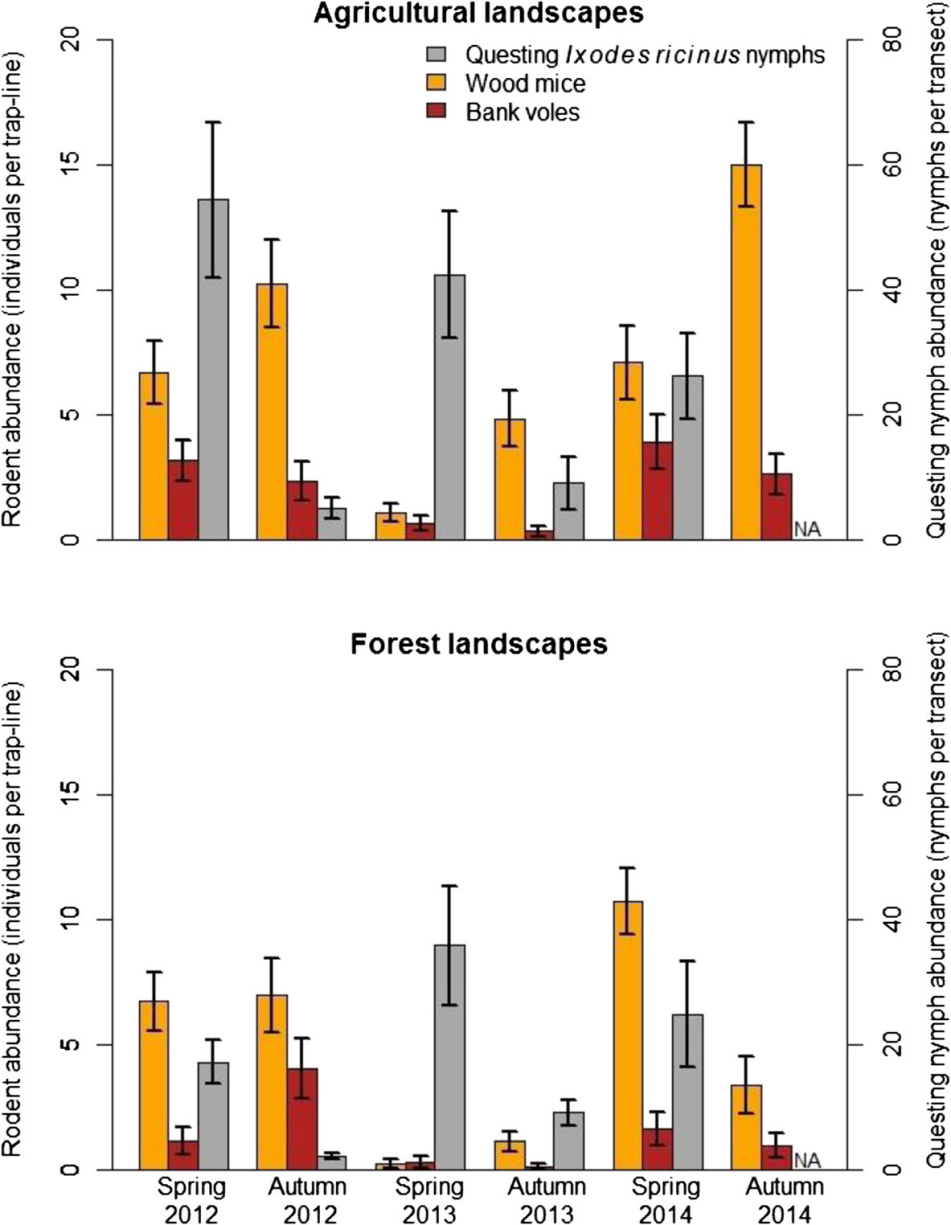
Abundances of rodent species and questing *Ixodes ricinus* nymphs. Abundances are shown for agricultural landscapes (*top panel*) and forest landscapes (*bottom panel*) for each sampling session (spring 2012 to autumn 2014) with standard errors

There were marked temporal variations in the abundances of small mammals over the 3 years of the study (Fig. 1). Higher small mammal abundances were observed in 2012 and 2014 (2012–2013: *V* = 297, *p* < 10^−4^; 2013–2014: *V* = 0, *p* < 10^−4^; 2012–2014: *V* = 111, *p* = 0.626). The abundance of all small mammals for 2012 and 2013 was significantly higher in autumn than in spring, but not in 2014 because of an unexpected population crash in the forest (2012: *V* = 78, *p* = 0.020; 2013: *V* = 40.5, *p* = 0.008; 2014: *V* = 138, *p* = 0.652).

There were significant spatial autocorrelations in small mammal abundance: for bank voles in spring 2012 (*I* = 0.043, *p* = 0.046) and wood mice in autumn 2013 and 2014 (*I* = 0.066, *p* = 0.010 and *I* = 0.161, *p* < 10^−4^, respectively). These results are probably due to high homogeneity of abundances in forest sites (numerous zero values) to which this method is sensitive [53]. The Moran’s I values are no longer significant when the agricultural landscapes and the forest landscape were analysed separately (*p* > 0.05). As (1) the analyses were conducted over 3 years and the spatial autocorrelation was not significant each season and (2) the significant Moran’s I values were weak and were not significant when the forest and agricultural landscapes were analysed separately, we assumed that these weak autocorrelations did not bias the results.

We tested the relationships between the wood mouse abundance and the bank vole abundance for the 6 combined sampling sessions, or by year (2012, 2013 and 2014), or by season (spring and autumn). For the 6 combined sessions, the abundances of the two species were significantly positively related (R^2^ = 0.129, *t* = 3.50 and *p* < 0.001), but not when years were considered separately (p-2012 = 0.669, p-2013 = 0.890 and p-2014 = 0.240). A significant positive relationship was found between the wood mouse abundance and the bank vole abundance in spring (R^2^ = 0.054, *t* = 2.00 and *p* = 0.049) and autumn (R^2^ = 0.109, *t* = 2.91 and *p* = 0.005). These results suggest that these two rodent species, which show annual synchronized fluctuations in abundance, do not compete strongly with each other because they differ in habitat use and feeding habits [42, 56, 57]. These two rodent species also differ in their resistance to ticks [22, 23, 33]. Therefore, it made sense to conduct separate analyses for these two rodent species with respect to the relationships between the landscape features, their abundance, their larval burden, the larval occurrence and the questing nymph abundance. Furthermore, no significant correlations were found between spring abundance and autumn abundance of wood mice (*t* = 0.808 and *p* = 0.419) and bank voles (*t* = −0.110 and *p* = 0.991), indicating that the abundances for each season can be considered as independent variables.

### Questing nymph abundance and larval occurrence index

A total of 4081 questing *I. ricinus* nymphs were collected: 3619 nymphs in the three springs and 462 nymphs in the two autumns (Fig. 1). There were 340 out of 1800 sub-transects where at least one larva was found (167 out of 1080 sub-transects in the three springs and 173 out of 720 in the two autumns). We also sampled 184 *I. ricinus* adults and 9 *I. frontalis* nymphs, but these ticks were excluded from the analyses. Questing nymphs were about 6 times more abundant in spring than in autumn (*V* = 35, *p* < 10^−12^) and questing nymph abundance in autumn was correlated to questing nymph abundance in spring (R^2^ = 0.13, *t* = 2.60 and *p* = 0.013). Nymphs in spring were significantly more abundant in 2013 than 2014 (*V* = 542, *p* = 0.001), almost significantly more abundant in 2012 than in 2014 (*V* = 411, *p* = 0.053), but no significant difference was observed between nymph abundance in spring 2012 and 2013 (*V* = 243, *p* = 0.241).

No autocorrelation was found for the number of questing *I. ricinus* nymphs when examining transects in the same habitat (*i.e*. excluding “paired transects”; *p* > 0.05). We found a spatial autocorrelation of larval occurrence index in spring 2012 in the wooded habitat (*I* = 0.063, *p* = 0.030) because of the high number of zero values in the forest landscape (13 out of 18), and because this method is sensitive to extreme values such as zeros [53]. For the same reason as for the small mammals, we assumed that these weak autocorrelations did not bias the results.

### Effects of the landscape features on small mammal abundances

A summary of the landscape models of small mammal abundance is shown in Table 2. In all the models for small mammal abundance, the year had an important influence on the abundance of wood mice (all *p*-values < 10^−13^) and bank voles all (*p*-values < 10^−5^).

**Table 2:** Small mammal abundances, *I. ricinus* larval occurrence and questing *I. ricinus* nymph abundance as a function of the landscape features

	Effect estimates (± SE) on wood mouse abundance: GLMPs			
Season	Spring		Autumn	
Landscapes	All	Agricultural	All	Agricultural
N plots	3 × 24	3 × 12	3 × 24	3 × 12
Intercept	2.01 (±0.075)***	1.82 (±0.116)***	2.07 (±0.172)***	2.32 (±0.090)***
Sampling year	***	***	***	***
EcoL		0.366 (±0.069)***		0.107 (±0.051)*
Wood	0.156 (±0.059)**	0.211 (±0.080)**	−0.361 (±0.062)***	
ENN-Wood			0.113 (±0.043)*	
	Effect estimates (± SE) on bank vole abundance effect: GLMPs			
Season	Spring		Autumn	
Landscapes	All	Agricultural	All	Agricultural
N plots	3 × 24	3 × 12	3 × 24	3 × 12
Intercept	0.625 (±0.147)***	1.04 (±0.170)***	1.15 (±0.115)***	0.823 (±0.191)***
Sampling year	***	***	***	***
EcoL	−0.410 (±0.113)***	−0.447 (±0.129)***		0.227 (±0.115)°
Wood	−0.645 (±0.110)***	−0.286 (±0.112)**	0.196 (±0.104)°	
ENN-Wood			0.224 (±0.095)*	
	Effect estimates (± SE) on larva occurrence index: GLMPs			
Season	Spring		Autumn	
Landscapes	All	Agricultural	All	Agricultural
N plots	3 × 24	3 × 12	2 × 24	2 × 12
Intercept	0.536 (±0.156)***	1.02 (±0.105)***	1.48 (±0.098)***	1.63 (±0.128)***
Sampling year	**		**°**	*
EcoL				
Wood		0.478 (±0.105)***		
ENN-Wood				
	Effect estimates (± SE) on questing nymph abundance: GLMNBs			
Season	Spring		Autumn	
Landscapes	All	Agricultural	All	Agricultural
N plots	3 × 24	3 × 12	2 × 24	2 × 12
Intercept	3.51 (±0.125)***	4.44 (±0.586)***	1.22 (±0.226)***	4.21 (±0.495)***
Sampling year			**	
EcoL	−0.232 (±0.127)			
Wood		1.13 (±0.692)°		3.92 (±0.870)***
ENN-Wood			−0.408 (±0.161)*	

Small mammal abundances, *I. ricinus* larval occurrence index and questing *I. ricinus* nymph abundance were modelled in generalized linear models of with the landscape variables and the sampling year as explanatory factors. The response variables were modelled using a Poisson error distribution for small mammal abundances and larval occurrence index (GLMPs) and a negative binomial error distribution (GLMNBs) for questing nymph abundance to account for the over-dispersion of the data. Each model contains all the significant explanatory variables (i.e. multiple regressions). The slope and standard error of the numeric variables from the model with the lowest AICc are given (see text). Significant codes are “°”: alpha = 0.1 “*”: alpha = 0.05, “**”: alpha = 0.01 and “***”: alpha = 0.001. Significant estimates (*p* < 0.05) are in bold

For all the landscapes combined, the wood mouse abundance had a significant positive relationship with the proportion of woodland in the landscape in the spring (*p* = 0.009) but a significant negative relationship in autumn (*p* < 10^−8^). For all the landscapes combined, there was a significant positive relationship between the wood mouse abundance in autumn and the mean distance between wooded patches (*p* = 0.010). In the agricultural landscapes, the wood mouse abundance in spring and autumn was significantly positively related to the length of the wooded habitat-grassland ecotone (p-spring < 10^−6^ and p-autumn = 0.040) and in spring with the proportion of woodland cover (*p* = 0.008).

For all the landscapes combined, there was a significant negative relationship of the bank vole abundance in autumn with the length of the wooded habitat-grassland ecotone (*p* < 10^−3^) and a significant negative relationship with the woodland cover (*p* < 10^−9^). For all the landscapes combined, the bank vole abundance in autumn was significantly positively related to the mean distance between wooded patches (*p* = 0.023). In the agricultural landscapes, the bank vole abundance in spring had also a significant negative relationship with the length of the wooded habitat-grassland ecotone (*p* < 10^−3^) and with the proportion of woodland cover (*p* = 0.008).

### Effects of the landscape features on questing immature ticks

A summary of the landscape models of immature ticks is shown in Table 2. The sampling year only had influenced the larval occurrence for all the landscapes combined in spring (*p* = 0.005) and in agricultural landscapes in autumn (*p* = 0.047). The larval occurrence index was significantly related to the proportion of woodland in the agricultural landscapes in spring (*p* < 10^−6^).

The sampling year only influenced the questing nymph abundance in all the landscapes in autumn (*p* = 0.008). In all the landscapes combined, the questing nymph abundance was significantly negatively related to the mean distance between wooded patches in autumn (*p* = 0.025). In the agricultural landscapes, the questing nymph abundance was significantly positively related to woodland cover in autumn (*p* < 10^−9^).

### Small mammal-immature tick relationships

#### Larval burden of small mammals

**Fig. 2:**
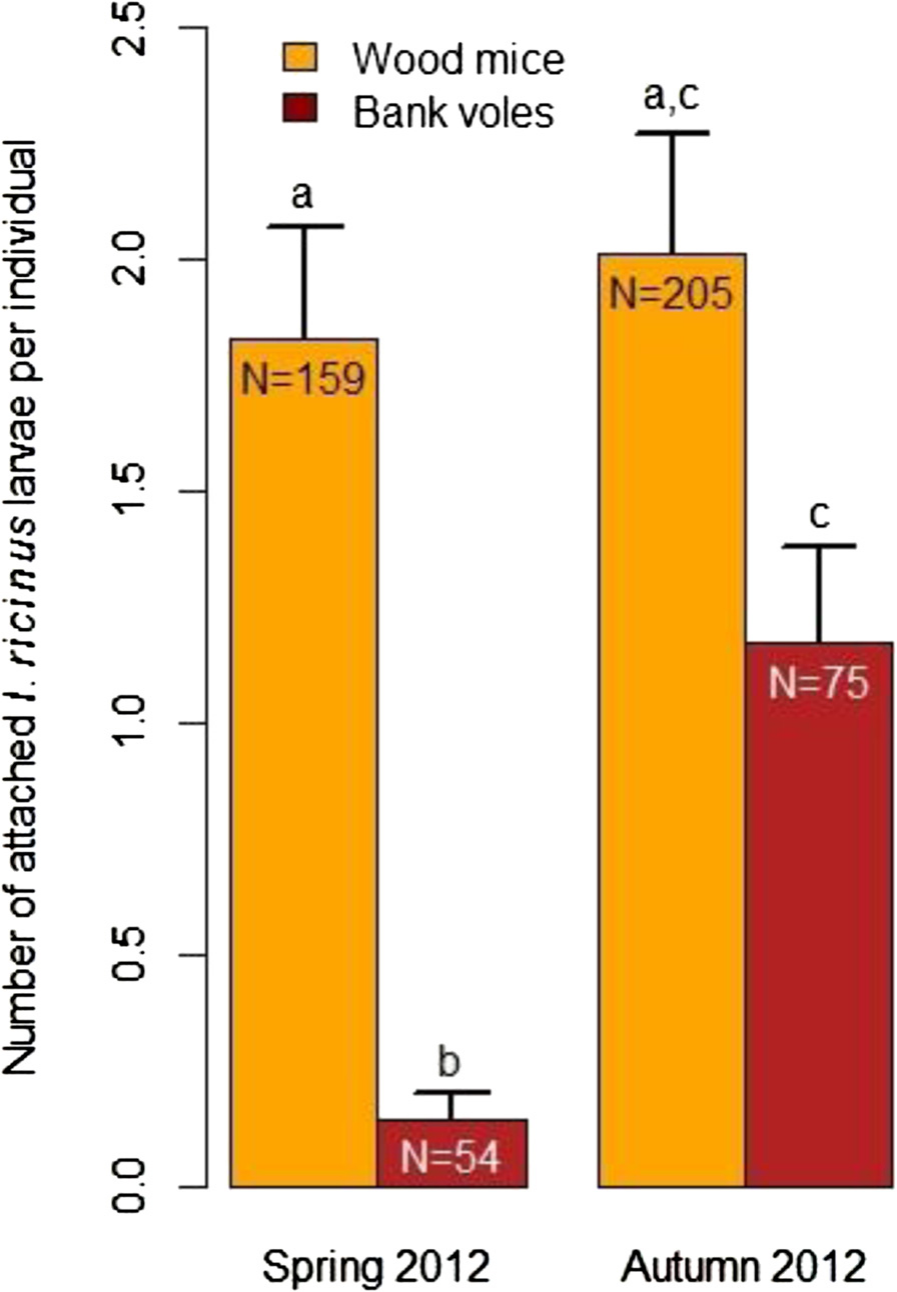
Average of *I*xodes *ricinus* larval burden per rodent species by season. The means with the standard errors of *I. ricinus* larval burden on wood mice and bank voles in spring and autumn 2012 are shown. Bars with the same letters (a, b and c) are not significantly different from each other (Mann–Whitney tests with *p* < 0.05). *N* = number of rodents considered for the analysis

The rodents trapped in 2012 carried a total tick burden of 802 *I. ricinus* larvae and 8 *Ixodes* sp. nymphs. In the spring, the larval burden on wood mice (1.83 larvae per wood mouse) was 12.4 times higher than that on bank voles (0.148 larvae per bank vole) and this difference was statistically significant (*W* = 2272, *p* < 10^−7^). In the autumn, the larval burden on wood mice (2.01 larvae per wood mouse) was 1.7 times higher than that on bank voles (1.17 larvae per bank vole), but this difference was not significant (*W* = 6905, *p* = 0.168). Finally, there was no significant difference in the mean *I. ricinus* larval burden of wood mice between seasons (*W* = 16,345, *p* = 0.960) whereas bank voles harbored significantly more larvae in autumn (*W* = 1208, *p* < 10^−5^). These results are shown in Fig. 2.

A summary of the relationships between the larval occurrence index and the larval burden on small mammals in 2012 is shown in Table 3. The total number of larvae on small mammals in spring and autumn was significantly positively correlated to the larval occurrence index. This result was also true for wood mice, but not for bank voles. In autumn, the larval occurrence index was significantly positively correlated with the mean number of larvae per small mammal and per wood mouse, but not per bank vole.

**Table 3:** *I. ricinus* larval burden of small mammals as a function of the larval occurrence index

	Total number of larvae attached on rodents per trap-line							
Host species	Spring				Autumn			
	Estimate (± SE)	R^2^	t	p	Estimate (± SE)	R^2^	t	p
Wood mice	**2.66 (±1.15)**	**0.194**	**2.30**	**0.031**	**4.12 (±1.91)**	**0.175**	**2.16**	**0.042**
Bank voles	−0.019 (±0.060)	0.004	−0.314	0.757	0.400 (±0.355)	0.054	1.13	0.271
Both rodent species	**2.64 (±1.16)**	**0.191**	**2.28**	**0.032**	**4.52 (±1.92)**	**0.202**	**2.36**	**0.028**
	Mean number of larvae attached per individual rodent per trap-line							
Host species	Spring				Autumn			
	Estimate (± SE)	R^2^	t	p	Estimate (± SE)	R^2^	t	p
Wood mice	0.185 (±0.125)	0.094	1.47	0.155	**0.335 (±0.154)**	**0.177**	**2.17**	**0.041**
Bank voles	0.010 (±0.042)	0.004	0.251	0.805	0.114 (±0.080)	0.102	1.43	0.170
Both rodent species	0.197(±0.141)	0.109	1.40	0.180	**0.474 (±0.185)**	**0.267**	**2.56**	**0.020**

Linear models of (1) the total number of attached *I. ricinus* larvae per trap-line on wood mice, bank voles and both rodent species and (2) the mean number of *I. ricinus* larvae per individual rodent per trap-line for wood mice, bank voles and both rodent species as a function of the larval occurrence index in 2012. Significant estimates are in bold (*p* < 0.05)

#### Effect of the small mammal larval burden on the questing nymph abundance the following year

The questing nymph abundance in spring 2013 was significantly correlated with the larval burden on small mammals in 2012 (larval burden was measured as the number of *I. ricinus*on small mammals per trap-line; spring: R^2^ = 0.183, *t* = 2.22, *p* = 0.037; autumn: R^2^ = 0.329, *t* = 3.28, *p* = 0.003). This correlation was also significant for wood mice in both seasons (spring: R^2^ = 0.189, *t* = 2.26, *p* = 0.034; autumn: R^2^ = 0.264, *t* = 2.81, *p* = 0.010) and weak but significant for bank voles in autumn (spring: R^2^ = 0.066, *t* = −1.25, *p* = 0.224; autumn: R^2^ = 0.168, *t* = 2.11, *p* = 0.046).

**Fig. 3:**
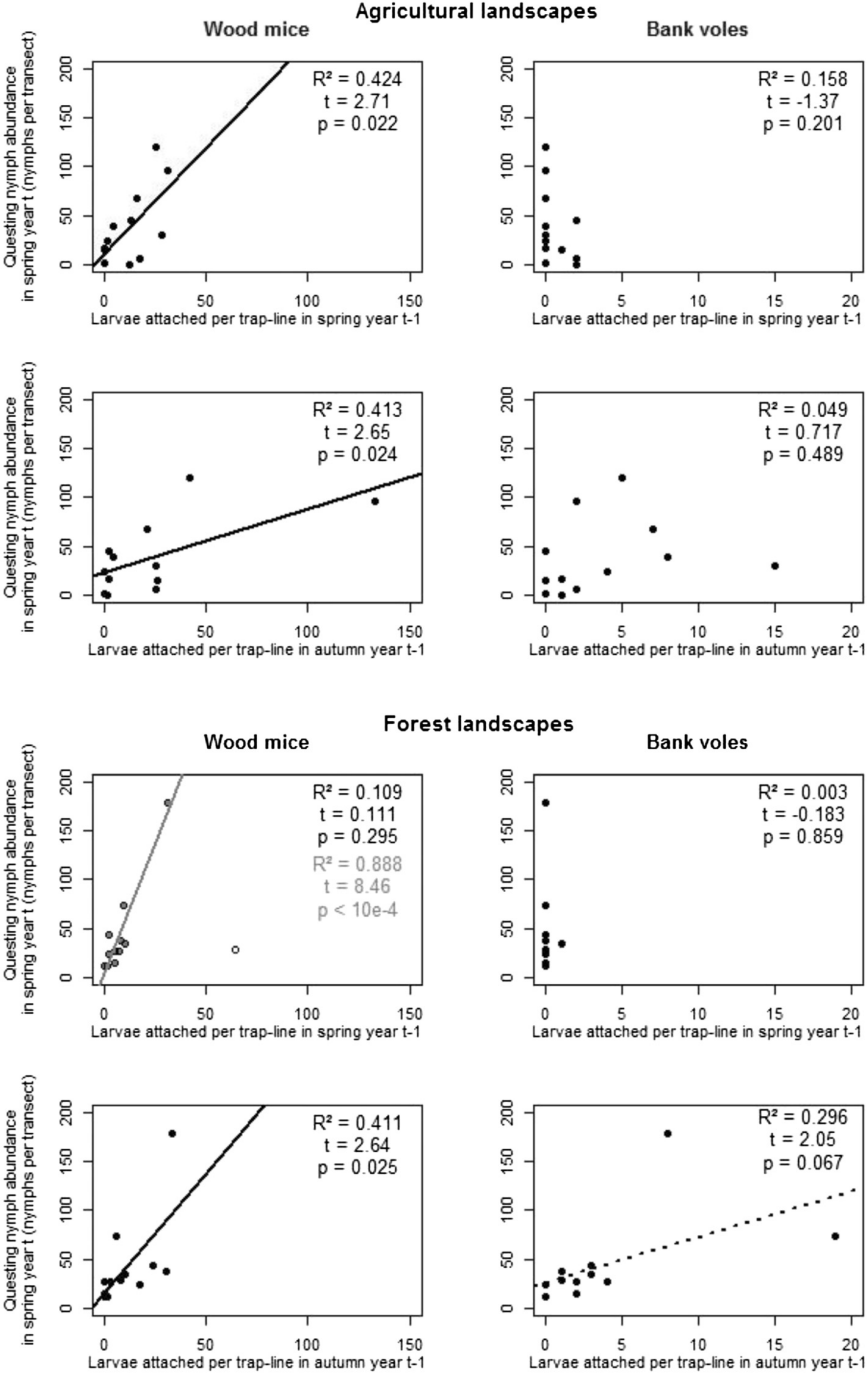
Correlations between the total *I. ricinus* larval burden of small mammal and the questing *I. ricinus* nymph abundance. The questing *I. ricinus* nymph abundance in the spring is modelled as a function of the total *I. ricinus* larval burden the previous year for wood mice on the left and for bank voles on the right, for the agricultural landscape (first and second rows) and the forest landscapes (third and fourth rows), in spring (first and third rows) and autumn (second and fourth rows). For each panel, the line of best fit from the simple linear regression (when significant), the R^2^ value and *p*-value are shown

By conducting separate analyses for the forest and agricultural landscapes, the correlations between the total number of larvae on small mammals and the abundance of questing nymphs the following year in the agricultural landscape in spring and in autumn were improved (spring: R^2^ = 0.383, *t* = 2.49, *p* = 0.032 and autumn: R^2^ = 0.440, *t* = 2.80, *p* = 0.019). This was true for wood mice, but not for bank voles (Fig. 3). In the forest, this correlation was not significant in spring for all small mammals (R^2^ = 0.113, *t* = 1.13, *p* = 0.285), wood mice, or bank voles. However, after removing an outlier site where numerous larvae were aggregated on a few wood mice, this correlation was significant for all small mammals (R^2^ = 0.890, *t* = 8.55, *p* < 10^−4^) and for wood mice (Fig. 3). In the forest in autumn, significant positive correlations were found for all small mammals (R^2^ = 0.614, *t* = 3.99, *p* = 0.003), for wood mice alone and for bank voles alone (Fig. 3).

#### Effect of small mammal abundance and questing larval occurrence on the questing nymph abundance the following year

A summary of the questing nymph abundance models as a function of the larval occurrence and small mammal abundance is shown in Table 4. There was a significant positive relationship between the larval occurrence index of each season and the questing nymph abundance the following spring. The questing nymph abundance was significantly positively related to the wood mouse abundance the previous spring and to the bank vole abundance the previous autumn.

**Table 4:** Questing *I. ricinus* nymph abundance as a function of the larval occurrence index and small mammal abundances

Explanatory variable	Abundance of *I. ricinus* questing nymphs in the spring of year t					
	Spring			Autumn		
	Estimate (± SE)	z-value	p	Estimate (± SE)	z-value	p
Larval occurrence index	**0.182 (±0.056)**	**3.24**	**<10** ^**−3**^	**0.151 (±0.066)**	**2.29**	**0.023**
Wood mouse abundance	**0.081 (±0.034)**	**2.41**	**0.011**	0.031 (±0.280)	1.13	0.272
Bank vole abundance	−0.065 (±0.074)	−0.878	0.411	**0.104 (±0.050)**	**2.09**	**0.028**
Rodent abundance	0.047 (±0.028)	1.68	0.080	0.043 (±0.021)	1.99	0.052

The abundance of questing *I. ricinus* nymphs in the spring of year t was modelled using generalized linear models and a negative binomial error distribution. Explanatory variables include: the larval occurrence index in year t-1, the abundance of wood mice in year t-1, the abundance of bank voles in year t-1, and the abundance of all rodents in year t-1. Each model contains only one explanatory variable (i.e. simple regression). The slope and standard error are shown for each one-variable model. Significant estimates are in bold (*p* < 0.05)

### *Integrative models to predict the questing* I. ricinus *nymph abundance*

The purpose of this statistical analysis was to determine which factors drive the abundance of questing nymphs. As nymphs were more abundant in spring and correlated to nymph abundance in autumn for which only 2 years of data were available, we modelled only the spring questing nymph abundance in the integrative models. We examined the importance of the 3 landscape variables, the 4 small mammal abundance variables (2 rodent species × 2 seasons), the larval occurrence index the previous spring and the sampling year in explaining the variation in the questing nymph abundance (Table 5). The four small mammal variables can be reasonably considered independent. We used the larval occurrence index of the previous spring rather than the larval occurrence index of the previous autumn, because it better explained the questing nymph abundance the following year (AICc-spring model = 427 and AICc-autumn model = 435) and the two indices were correlated (constrained linear model: R^2^ = 0.586, *t* = 8.15, *p* < 10^−9^).

**Table 5:** Questing *I. ricinus* nymph abundance as a function of the *I. ricinus* larval occurrence index, small mammal abundance and landscape features

	Effect estimates (± SE) on questing *I. ricinus* nymph abundance: GLMNBs		
Landscapes	All landscapes	Agricultural	Forest
Sub-model (included variables)	All variables	All variables	Landscape excluded
N transects	2 × 36	2 × 18	2 × 18
Intercept	3.26 (±0.117)***	3.11 (±0.194)***	3.28 (±0.093)***
Sampling year			
Recruitment variable			
Larva occurrence index spring t-1	0.325 (±0.129)*	0.540 (±0.219)**	0.513 (±0.090)***
Host variables			
Wood mouse abundance spring t-1	0.417 (±0.135)**		
Wood mouse abundance autumn t-1			
Bank vole abundance spring t-1	−0.351 (±0.126)**		
Bank vole abundance autumn t-1			0.446 (±0.091)***
Environmental variables			
EcoL	−0.500 (±0.125)***		-
Wood		0.355 (±0.224)	-
ENN-Wood			-

Generalized linear models of the abundance of questing *I. ricinus* nymph abundance in spring in the agricultural landscapes, the forest landscapes, and all the landscapes combined. The response variable was modelled using a negative binomial error distribution (GLMNBs). The explanatory variables include the larval occurrence index the previous year (in spring and autumn), the rodent abundance the previous year (in spring and autumn), the landscape variables (except in the forest landscapes models) and the sampling year. Each model contains all the significant explanatory variables (i.e. multiple regressions). The slope and standard error of the numeric variables from the model with the lowest AICc are given (see text). Significant codes are “°”: alpha = 0.1, “*”: alpha = 0.05, “**”: alpha = 0.01 and “***”: alpha = 0.001. Significant estimates (*p* < 0.05) are in bold

In the simplified models for the landscapes combined, the agricultural landscapes, and the forest landscapes, the questing nymph abundance was significantly positively related to the larval occurrence index of the previous spring (*p* = 0.014, *p* = 0.003 and *p* < 10^−11^ respectively). In the landscapes combined model, the questing nymph abundance was significantly positively related to the wood mouse abundance the previous spring (*p* = 0.002), significantly negatively related to the bank vole abundance in the previous spring (*p* = 0.015) and significantly negatively to the length of the wooded habitat-grassland ecotone (*p* < 10^−3^). In the agricultural landscapes model, the best model included the woodland cover, but this landscape variable did not have a significant effect on the questing nymph abundance (*p* = 0.077). In the forest landscapes model, the questing nymph abundance was significantly positively related to the bank vole abundance the previous autumn (*p* < 10^−7^).

## Discussion

This study provides an overview of small mammal-*Ixodes ricinus* tick interactions in agricultural landscapes and a neighboring deciduous forest. The small mammal community was dominated by two rodent species: the wood mouse and the bank vole, which accounted for 76.5 and 22.3 % of the 1145 captured small mammals. Our sampling covered 3 years of rodent population abundances in spring and autumn, showing strong inter and intra-annual variations, and 3 years of questing tick abundance. This temporal variation in host abundance explained a substantial portion of the variation in the abundance of questing nymphs the following year. We also found variations of the host-parasite relationship in the two types of landscapes.

### Influence of the landscape features on small mammals

The landscape features had various effects on the abundance of small mammal species. In general, habitat loss and habitat fragmentation are unfavourable to specialist species, but favorable to generalist ones [58]. In fact, the effects of habitat fragmentation depend on the species and their interactions [4, 31, 59]. A higher small mammal density in woodland-fragmented landscapes may be caused by reduced interspecific competition and/or reduced predation because of reduced landscape connectivity and/or edge effects for wood specialist species [59–61]. Such a positive response was observed for the bank vole. Indeed, the abundance of the bank voles was positively related to our measure of woodland fragmentation (ENN-Wood) and negatively related to woodland cover, which is negatively related to woodland fragmentation.

A counter-intuitive response to these landscape features was observed for wood mice. In agricultural landscapes, while the bank voles remain in the hedgerows and woods, the wood mouse density in these habitats decreases in spring because a significant portion of the wood mouse population disperses into the surrounding crop fields [42, 56, 62]. As fewer individuals disperse into crops in woodland-dominated landscapes, this wood mouse dispersion leads to an apparent positive effect of woodland cover on wood mouse abundance at this season. In autumn, after the harvest, wood mice return ‘en masse’ from the fields to the hedgerows and woods inducing a suddenly high population density in these habitats of crop-dominated landscapes [42, 56, 62]. When agricultural and forest landscape sites are considered together, this high density of wood mice in crop-dominated landscapes leads to an apparent negative effect of the woodland cover on wood mouse abundance and a positive effect of woodland fragmentation.

The length of the wooded habitat-grassland ecotone explained a substantial part of the variation of small mammal abundance in the agricultural landscapes. This variable had a positive effect on the wood mouse abundance in spring and autumn, but was negatively related to the bank vole abundance in spring. These ecotones are rich permanent habitats, which provide food and shelter throughout the year [42, 63]. When associated with linear structures like hedgerows in agricultural landscapes, they enhance the connectivity between patches of suitable wooded habitat [63, 64]. Alternatively, bank vole density can be higher in isolated patches, possibly explaining the negative relationship between the abundance of this species and the length of the wooded habitat-grassland ecotone and with the woodland cover [46].

### *Influence of the landscape features on immature* I. ricinus *ticks*

Larval and nymphal ticks were related to landscape features probably because of indirect effects of host density and host behaviour. The larval occurrence index was positively related to the proportion of woodland cover in agricultural landscapes. This positive effect can be explained by the fact that the woodland cover is favourable to roe deer, which are the main hosts of female ticks [65, 66]. As larvae have poor dispersal ability, one expects to find larvae in habitats where hosts of female ticks spend a lot of time. Female ticks that drop off their vertebrate host in these habitats will therefore lay their eggs in these habitats.

The positive relationship between questing nymph abundance and woodland cover in the agricultural landscapes was probably driven by the fact that this landscape variable is also positively related with the larval occurrence index and wood mouse abundance (see the next section). The analysis of the landscape features found no significant effect of the length of the wooded habitat-grassland ecotone on the abundance of questing nymphs (Table 2). In contrast, the integrative models showed that the length of wooded habitat-grassland ecotone had a significant negative effect on the abundance of questing nymphs (Table 5). This variable is linked to grassland cover which is used as pastures for cattle or mowed regularly, and is therefore not suitable for ticks (trampling, short vegetation). Additionally, when cattle are present, they encounter nymphs mostly at the border of the pasture, but engorged nymphs may often detach in the centre of the pasture. This phenomenon may lower the number of questing nymphs without amplifying the tick abundance and may explain the negative effect of wooded habitat-grassland ecotone, despite an indirect positive effect of wood mouse abundance (see next section).

### *Relationships between small mammal abundance,* I. ricinus *larval occurrence, small mammal* I. ricinus *larval burden and questing* I. ricinus *nymph abundance*

Our study supports our first hypothesis that the occurrence of larval ticks was a good predictor of the abundance of questing nymphs the following year and of the total larval burden of small mammals. This finding confirms the importance of larval occurrence (i.e. the location where female ticks lay their eggs) in the population dynamics of this tick species, particularly in the agricultural landscapes. In addition, the abundance of questing nymphs in spring was positively related to the wood mouse abundance in the previous spring and the bank vole abundance in the previous autumn in the forest. The importance of small mammals for feeding larval ticks is confirmed by the positive relationship between the questing nymph abundance in 2013 and the total larval burden on small mammals in 2012. Although a positive relationship between larval occurrence and questing nymph abundance is expected regardless of the host used by the larvae (our first hypothesis), the strong positive relationships between larval burden on small mammals and questing nymph abundance the following year supports our second hypothesis that small mammals play a major role in feeding larval ticks. Nevertheless, further studies are needed to provide more robust evidence. For instance, reliable blood meal analyses [67] or investigation on the genetic relatedness of questing larvae, larvae found on small mammals and questing nymphs the following year.

There was substantial variation in the tick burden of small mammals. Wood mice carried ten times more larvae than bank voles in spring, and two times more larvae than bank voles in autumn, but this difference was significant only in the spring. Numerous studies have shown that wood mice carry more larval ticks than bank voles [39, 68–71]. Bank voles, but not *Apodemus* mice, are able to develop an acquired resistance to ticks after successive infestations [22, 24, 33] and this phenomenon can explain the difference in tick burden between these two rodent species. In summer and autumn, bank vole populations consist mostly of young individuals that are susceptible to ticks. In spring by contrast, bank vole population consists mainly of overwintering adults. These adult bank voles were likely already exposed to ticks and developed their resistance to ticks the previous year. Our results support this hypothesis. First, bank voles had 8 times more ticks in autumn than in spring, and second, the larval occurrence index was not correlated to the mean number of larvae attached on bank voles, suggesting that there is variation in susceptibility among individuals. Thus, seasonal changes in the age structure of bank vole populations may have important implications for larval ticks.

The peak larval burden on small mammals is in late spring [39]. The larval ticks are found in the hedgerows and woodlots and not in the surrounding crop fields because the latter is a hostile environment for tick survival (absence of humus and leaf litter, sun, wind, and pesticide exposure). In the isolated hedgerows and woodlots of agricultural landscapes, bank voles may be more abundant because a part of the wood mouse population disperses into crop fields. This seasonal pattern is unlikely to be caused by bank vole dominance, as we found no evidence of competition between these two species. Thus the bank voles could encounter a large proportion of the larval ticks and develop resistance to ticks resulting in a negative effect on the nymph abundance the following year.

Our results show the complexity of host-tick relationships [72]. No effect of the wood mouse abundance in autumn on the abundance of questing nymphs was observed. The bank voles, by drawing larvae away from wood mice, could reduce the effect of wood mice when the two species are sympatric [73]. However, the total larval burden on wood mice per trap-line, which was positively correlated to the larval occurrence index, was positively correlated to the questing nymph abundance the following year. This finding is consistent with the tolerance of the wood mouse to tick attachment, as the mean larval burden per wood mouse per trap-line is itself correlated with the larval occurrence index. These results confirm our third hypothesis, small mammal species differ in their contribution to the abundance of *I. ricinus* nymphs and wood mouse play a key role in the population dynamics of *I. ricinus* in Western Europe [39, 70].

### Epidemiological implications for tick-borne diseases

Our results confirm our fourth hypothesis: woodland and wooded habitats-grassland ecotones in agricultural landscapes are suitable habitats for ticks. We found no positive effect of woodland cover on questing nymph abundance when considering all the landscapes, whereas ticks were expected to be more numerous in forest landscapes. These results question the role of forest as the predominant source of ticks [37, 43] and suggests that small fragments of suitable habitat in agricultural landscapes (copses and hedgerows) could also be important for the maintenance of tick populations. Wooded habitats in agricultural landscapes can be favourable for *I. ricinus* tick populations because they create isolated patches with high densities of small mammal hosts [30]. Alternatively, small fragments of wooded habitats can be important in maintaining the landscape connectivity and the host diversity and thus dilute, or amplify, tick-borne pathogens, depending on the competence of the hosts in the community. Further studies should include prevalence data on tick-borne infectious agents to better understand the effect of landscape structure on their transmission.

Our study found a difference in tick ecology between the forest and agricultural landscape because rodent host species had a different effect on the nymph abundance. Small mammals are important reservoir hosts of infectious agents responsible for diseases of humans and livestock. One could expect a different transmission and prevalence of infectious agents in ticks in the landscape according to the structure of the small mammal community. For instance, wood mice and bank voles differ in their reservoir competence for *B. afzelii* and *B. microti*. One can thus expect a higher prevalence of *B. afzelii* in ticks from landscapes where wood mice are abundant and of *B. microti* in ticks from landscapes where bank voles are abundant.

We trapped small mammals only in wooded habitats (woods and hedgerows), but it has also been shown that rodent communities in different habitats can vary in species composition and harbor different communities of tick species [16]. For instance, Welc-Falęciak and colleagues [29] showed that *I. ricinus* was more abundant on rodents from wooded habitat (bank voles and yellow-necked mice) whereas *Dermacentor reticulatus* ticks were more abundant on rodents from fallow land (*Microtus* voles). They suggested a possible implication of these various rodent species on the enzootic maintenance of *B.microti*. Some authors have already investigated the factors that influence the prevalence of tick-borne pathogens in ticks in pasture [48] or in woodland versus pasture [74], but without accounting for the landscape context. Other studies have considered different host species at a regional scale (domestic and wild ungulates [65, 75]) or meteorological, climatic or microclimatic conditions, which are relevant to explain tick activity and development [10, 41].

Our study relies on the assumption of a 1-year delay between larval and nymphal stages of ticks (from spring or autumn to the next spring), but, actually, this may be more or less than 1 year. Additionally, some factors could have biased our abundance estimates of ticks and small mammals, which are: (1) differing site characteristics like vegetation structure [46, 76, 77], (2) the abundance of other hosts (e.g. medium-sized mammals, ungulates and birds [14, 15, 78]), and (3) different agricultural practices and forest management. Accounting for all these factors in the same study is unrealistic, but they should be accounted for in future investigations.

The heterogeneity and the dynamics of land cover composition and configuration in agricultural landscapes, which influences the community of vertebrate hosts, makes these host-vector-pathogen systems more complex in agricultural ecosystems. It would be interesting to investigate more deeply the temporal and spatial variation of tick densities in agricultural landscapes at different scales. Previous studies have shown that the woodland connectivity in the landscape influence the occurrence and abundance of ticks at a regional scale [79, 80]. Further studies on the influence of the landscape on the transmission of tick-borne diseases are needed. A possible approach to better understand the ecology of tick-borne diseases would be to evaluate the influence of the functional connectivity at the spatial scale of the home range of various hosts (small mammals, roe deer or territorial ground-feeding birds) on the prevalence and diversity of infectious agents occurring in tick populations. We are currently conducting such a study.

## Conclusion

This study shows that the larval occurrence is a determinant factor of the abundance of *I. ricinus* nymphal ticks. Our results showthat wood mice, which dominate the small mammal community, are a driver of the population dynamics of *I. ricinus* tick populations in the agricultural landscapes of temperate Western Europe. As wood mice feed a substantial proportion of larval ticks, they likely play a key role in the transmission of tick-borne diseases. The acquired resistance to ticks in the bank vole may reduce the role of this species as a trophic resource over time. The woodland cover has a positive effect on the larval tick occurrence, probably by favouring the local abundance of the vertebrate hosts of adult ticks (roe deer). Woodland cover and connectivity have an indirect positive influence on the abundance of nymphal ticks by favouring wood mouse abundance. Our study has important epidemiological implications for the transmission of tick-borne diseases.

## Figures and Tables

**ᅟ:**
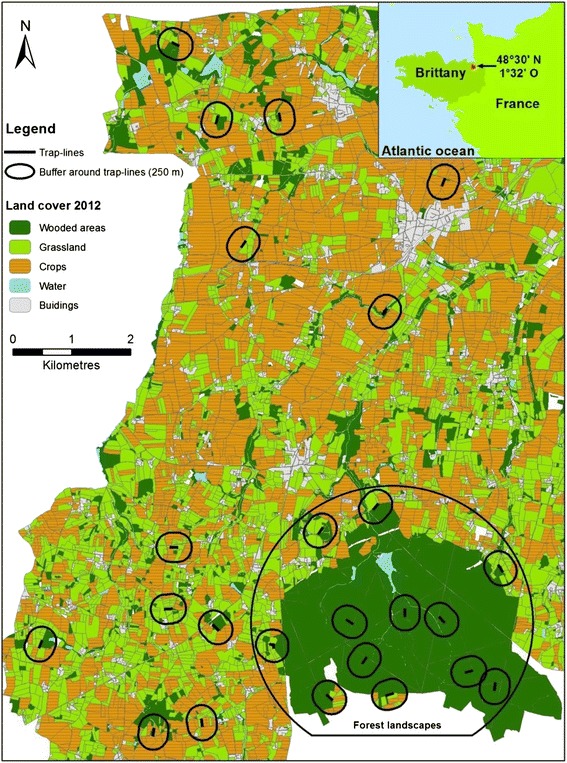
ᅟ

## Appendix 1

Land cover 2012, sampling locations with related buffers (12 in agricultural landscapes and 12 in forest landscape) in the “Zone atelier Armorique” (Brittany, France).
